# Relationship between Burnout and Mental-Illness-Related Stigma among Nonprofessional Occupational Mental Health Staff

**DOI:** 10.1155/2019/5921703

**Published:** 2019-09-24

**Authors:** Tomoe Mitake, Shinichi Iwasaki, Yasuhiko Deguchi, Tomoko Nitta, Yukako Nogi, Aya Kadowaki, Akihiro Niki, Koki Inoue

**Affiliations:** Department of Neuropsychiatry, Osaka City University, Graduate School of Medicine, 1-4-3 Asahimachi Abeno-ku, Osaka 545-8585, Japan

## Abstract

**Background:**

Stigma related to mental illness can be an obstacle affecting the quality of life of people with mental illness. Although mental illness in the workplace is a public problem globally, few studies have investigated the effect of stigma on job-related problems such as burnout.

**Aim:**

This study aimed to clarify the association between mental-illness-related stigma and burnout among nonprofessional occupational mental health staff.

**Methods:**

In this cross-sectional study, nonprofessional occupational mental health staff's perceived mental-illness-related stigma was assessed using Link's Devaluation-Discrimination Scale, and their burnout was assessed using the Maslach Burnout Inventory. The association between stigma and burnout was analyzed by multiple linear regression analysis.

**Results:**

In total, 282 participants completed the questionnaire (response rate: 91.3%). We excluded 54 nurses from the analysis to examine strictly nonprofessional occupational mental health staff. Finally, 228 eligible respondents were surveyed. Multiple linear regression analysis revealed that mental-illness-related stigma was significantly associated with a high degree of depersonalization, which was one of the burnout dimensions. However, the impact of stigma over the depersonalization domain of burnout was minor.

**Conclusion:**

The results suggest that higher perceived mental-illness-related stigma is associated with more severe burnout. It is important to take measures against mental-illness-related stigma to avoid burnout among occupational mental health staff.

## 1. Introduction

Stigma is the misrecognition or unfounded recognition of individuals or groups with specific attributes. In the past, such attributes have included skin color, race, sexual preference, infectious disease, and mental illness. Stigma is a serious social problem with negative consequences such as disadvantage, exclusion, and inequality for the targeted person or group. Stigma related to mental illness can be an obstacle affecting the quality of life of people with mental illness. Stigmatizing attitudes toward mental illness have been shown to harm the self-esteem of people suffering from mental illnesses [1], preventing them from seeking mental health services [2] and negatively affecting their employment opportunities [3].

Mental illness in the workplace is a global public problem. Because of the increasing number of employees with mental health issues in Japan, the “Guidelines for Maintaining and Improving Workers' Mental Health” was published in 2006 [4]. According to this guideline, employers are obligated to appoint occupational mental health staff. Occupational mental health staff members are in charge of practical mental health care in the workplace. Both mental health professionals (e.g., nurses, health nurses, and health supervisors) and nonprofessionals (e.g., labor management staff) may be allocated this position in Japan.

Previous studies have found work associated with mental health to be more stressful than work in other medical fields [5], and mental health professionals are at a high risk for developing burnout syndrome [6]. There are several studies of burnout focusing on mental health professionals. A study conducted in Wales indicated that one in two mental health nurses are emotionally overextended and exhausted by their work [7]. Mental health social workers in England and Wales showed significant symptomatology and distress associated with burnout twice the level of that reported by a similar survey conducted among psychiatrists [8]. Psychiatrists showed high levels of emotional exhaustion and depersonalization and had higher levels of burnout than other physicians [9]. Therefore, occupational mental health staff may often experience higher stress and burnout than people in other professions.

In Japanese workplaces, labor management staff, who are not mental health professionals, are often allocated occupational mental health staff roles. Due to their lack of knowledge of mental health issues, they are more likely to have a stigmatized view of mental illness. The behavioral impact of such stigma may include avoidance [10] of employees with mental illness. Mental-illness-related stigma can be understood as a possible cause of burnout.

We propose that the health of occupational mental health staff is deeply connected with employees' health. However, no study has evaluated the association between mental-illness-related stigma and burnout for nonprofessional occupational mental health staff. Therefore, the aim of this study was to clarify the association between this stigma and burnout for nonprofessional occupational mental health staff using self-report questionnaires. We hypothesized that higher perceived mental health stigma would be associated with more severe burnout among nonprofessional occupational mental health staff.

## 2. Methods

### 2.1. Subjects

In this cross-sectional study, we distributed self-administered anonymous questionnaires to 309 participants of mental health seminars for occupational mental health staff between 2017 and 2018. These seminars are conducted in accordance with the curriculum published by the Ministry of Health, Labor, and Welfare, and they include mental-health-related matters such as formulating mental health plans, responding to mental illness, providing support for returning to the workplace, and improving the workplace environment. Participants in these seminars voluntarily applied through a public online application. They were occupational mental health staff members, both mental health professionals and nonmental health professionals. Ten seminars were held in the Kansai region of Japan; 282 participants completed the questionnaire (response rate: 91.3%). We excluded 54 mental health professionals from the analysis to examine only non-mental health professionals. This was because we considered their knowledge of mental illness to be different from that of occupational mental health staff, and such differences may affect burnout and stigma.

All participants gave informed consent to participate as volunteers, and they understood that there was no penalty for choosing not to participate. Prior to the seminars, the participants provided the information described below.

### 2.2. Demographic Data

Demographic data collected in this study included participants' age and gender and the industry they worked in.

### 2.3. Measurement of Stigma

Stigma toward people with mental illness was measured by Link's Devaluation-Discrimination Scale (DDS) [11]. The reliability and validity of the Japanese version of this questionnaire have been previously established [12, 13]. DDS is a 12-item instrument that asks about the extent of agreement with statements indicating that “most people devalue current or former psychiatric patients by receiving them as failures, as less intelligent than other persons, and as individuals whose opinions need not be taken seriously” [14, 15]. The scale uses a 4-point Likert-type scale ranging from “agree” (4) to “disagree” (1) to measure the stigma. A higher score on the scale indicates a stronger perception of stigma, and reverse scoring was used where necessary. Cronbach's alpha coefficient was 0.80.

### 2.4. Measurement of Burnout

The Maslach Burnout Inventory (MBI) is the most widely used measure to assess burnout. We used the Japanese version of this scale [16, 17], and its reliability and validity were confirmed [18]. The MBI includes three burnout dimensions: emotional exhaustion (EE; depletion of emotional resources and feelings of fatigue), depersonalization (DP; negative, cynical attitude, and feelings about clients), and personal accomplishment (PA; self-evaluation of one's job effectiveness with regard to working with clients) [8]. The scale contains 17 items and uses a 5-point Likert-type scale ranging from “always yes” (1) to “no” (5) to measure EE (5 items), DP (6 items), and PA (6 items). Higher scores for EE and DP and a lower score for PA indicate a high tendency for burnout [19]. Cronbach's alpha coefficient for each dimension was as follows: 0.80 (EE), 0.82 (DP), and 0.79 (PA).

### 2.5. Ethics Statement

The Human Subjects Review Committee of Osaka City University approved the protocol of this study (authorization number: 1409). All data were stored only in our database, and the employer did not have access to the data or knowledge of who had participated in the study.

### 2.6. Statistical Analysis

An independent *t* test was used to examine the differences in age and gender. The distributions of the scores of burnout dimensions and the DDS were not normal. Therefore, a Mann–Whitney *U* test was used to identify the differences between the scores of burnout dimensions and DDS for gender. Similarly, the correlation between stigma and burnout was analyzed using Spearman's correlation. We performed a hierarchical multiple regression analysis to determine whether DDS explained the score of each burnout dimension. Gender and age were entered in step 1, and DDS was entered in step 2. Differences were considered significant at *p* < 0.05. All statistical analyses were performed using SPSS version 24.0 software (SPSS Inc., Chicago, IL).

## 3. Results

### 3.1. Subjects' Characteristics

Table 1 shows the subjects' characteristics and the mean scores for each burnout dimensions and DDS. The mean age of the study population was 46.2 ± 9.9, with that of male and female participants being 49.2 ± 9.2 and 43.2 ± 9.7, respectively. The mean age of the male participants was significantly higher than that of the female participants. The sample comprised 109 (47.8%) males and 119 (52.2%) females.

We found no differences in gender in the mean scores on each of the burnout dimensions and DDS, as shown by the Mann–Whitney *U* test. The number of persons involved in the manufacturing industry was the largest (34%); the second biggest industry was services (13%), which was followed by construction (9%).

### 3.2. Correlations between Stigma and Burnout

Table 2 shows Spearman's correlation between burnout dimensions and stigma according to DDS. There was a weak positive correlation between DP and stigma. All the burnout dimensions correlated with each other.

### 3.3. Multiple Linear Regression Analysis Examining the Associations between Stigma and Burnout

Table 3 shows the results of the hierarchical multiple linear regression analysis of the scores for each of the burnout dimensions and the scores for DDS. In step 1 of the analysis for EE, the results showed no significant predictor. In step 2, DDS accounted for no change in variance (*F* = 3.80, ns). In step 1 of the analysis for DP, there was no significant predictor. In step 2, DDS accounted for a significant additional 2.2% of the variance (*F* = 2.96, *p* < 0.05) (i.e., stigma predicted a higher level of DP). In step 1 of the analysis for PA, age was a significant predictor. In step 2, DDS accounted for no change in variance (*F* = 1.82, ns).

## 4. Discussion

In this study, we evaluated the association between mental-illness-related stigma and burnout using DDS and MBI measures. The participants were nonprofessional occupational mental health staff in a workplace. This study found that mental-illness-related stigma was significantly associated with DP. However, the impact of stigma over DP was minor. EE and PA were not associated with stigma.

To our knowledge, no prior studies have evaluated the association between mental-illness-related stigma and burnout dimensions among mental health nonprofessionals. Thus, the present results are discussed in the context of previous results obtained for mental health professionals. The burnout dimensions found to be correlated with mental-illness-related stigma have differed across various studies. One previous study conducted among mental health professionals found that a lower level of PA was associated with avoidant attitudes toward patients [20]. Another study found that all three burnout dimensions were correlated with mental health professionals' negative feelings toward patients [21]. The current study, meanwhile, revealed that mental-illness-related stigma had a significant effect only on DP but not on other burnout dimensions.

We consider several reasons for the differences in these results. First, our participants were nonprofessional occupational mental health staff (e.g., labor management staff), whereas in the previous studies, the participants were mental health professionals. As the number of employees suffering from mental illnesses in Japan is increasing, the demand for occupational mental health staff is increasing. However, in practice, in Japan, small-scale companies with less than 50 workers are often not able to secure a mental health professional, and nonprofessional staff are often appointed to occupational mental health roles [22]. Since they have little knowledge of mental health issues and are not familiar with appropriate responses to employee mental illness, this lack of expertise might affect which burnout dimensions are associated with stigma.

Second, stigma is a multidimensional phenomenon that can be subcategorized into self-stigma, public stigma, and stigmatizing experiences [23], and there are various measures to assess mental-illness-related stigma [24]. The differences between the scales measuring stigma might lead to different results. We used DDS to assess stigma because its validity and reliability are well established. In the future, we can get more comprehensive results by using various measures of stigma and comparing them.

Previous studies that examined the effects of mental-illness-related stigma used the social distance scale to measure the attitudes and behavior of individuals suffering from mental illness [25]. Mental-illness-related stigma may create a tendency to increase social distance from someone with mental illness. In our study, we found an association between mental illness stigma and DP. DP refers to negative, cynical, or excessively detached responses to various aspects of a job [26]. Increasing the preference for social distance might appear as DP. At the same time, in the present study, stigma was found not to be correlated with EE and PA. This could be because the preference for social distance might lead to the avoidance of communication with someone suffering from mental illness. Because of the absence of emotional communication, stigma might not affect EE. Additionally, our participants were nonprofessionals, and they had little knowledge of mental illness, so it was understandable that they did not feel PA regardless of the presence or absence of stigma.

In the present study, mental-illness-related stigma was associated with burnout. As such, the results suggest a need for antistigma or antiburnout interventions to support employees' mental health in Japanese workplaces. Such interventions for stigma and burnout could work complementarily. Reducing mental-illness-related stigma is a global issue, and many interventions attempting to reduce it have been conducted. The World Psychiatric Association's Global Antistigma Program was initiated in 2001 in six German cities. After three years, a representative of the association conducted a telephone survey in these cities (*N* = 4622) that confirmed the efficacy of the antistigma intervention [27]. The results of a previous systematic review indicated that social contact is the most effective type of intervention for improving stigma-related knowledge and attitudes in the short term (<4 weeks) [28]. In Japan, systematic reviews regarding antistigma interventions among university and college students indicated that social contact and video-based social contact may improve attitudes toward mental illness and reduce social distancing from people with mental illnesses [29]. It is suggested that in the workplace, mental health education focusing on social contact for occupational mental health staff may be useful for reducing stigma.

Furthermore, we should recognize the need to intervene in burnout among occupational mental health staff. Previous studies have examined the effectiveness of a number of burnout intervention programs in workplaces. Cognitive behavioral training and counseling as well as adaptive coping with refresher courses have been shown to decrease burnout [30, 31]. We should focus on both the risk of burnout and mental-illness-related stigma, which make the work of occupational mental health staff difficult to address.

The effect size of the association between DP and stigma was small. Previous studies have examined the correlation between burnout and stigma among mental health professionals. For example, a sample of mental health professionals showed a weak correlation between lower levels of PA and stigmatizing attitudes [20]. In another study, a moderate correlation was found between burnout and negative feelings toward patients among psychiatric staff [21]. A possible explanation for these moderate or weak correlations between burnout and stigma is that other variables might affect or mediate the outcomes. Therefore, future research should account for other variables such as mental health problems, self-esteem, and personal contact with people with mental health problems.

This study had several limitations. First, the small sample size might limit the generalizability of the findings. Second, we used self-reported questionnaires; therefore, the results may be influenced by personal differences or response tendencies. Third, this was a cross-sectional study; thus, it cannot be inferred with certainty from our data that the relationship between stigma and burnout is causal. Fourth, differences in work-related status (e.g., occupation, stage of career, industry, salary, hours worked, and company size) could potentially bias the results. The size of the company participants worked for ranged from several people to twenty thousand people. The industry types and the positions held also differed. These potential biases might have influenced the interpretation of the results. Further study is needed to compare the results for mental health professionals and nonprofessionals working for relatively large companies with those working for relatively small companies. Finally, our data were collected from participants in mental health seminars. Therefore, we must consider a potential selection bias. To avoid this bias, a study based on random sampling should be conducted in the future. Future cohort or longitudinal research addressing the relationship between stigma and burnout in the workplace might also be beneficial.

## 5. Conclusion

This is the first study showing that mental-illness-related stigma is significantly associated with the burnout dimension of depersonalization among nonprofessional occupational mental health staff in Japan. The results suggest the importance of efforts to reduce mental-illness-related stigma in the workplace. To avoid burnout among occupational mental health staff, it is important to take measures against mental-illness-related stigma.

## Figures and Tables

**Table 1:** Participant demographic variables, Maslach Burnout Inventory scores, and Devaluation-Discrimination score.

	Mean ± SD			
	Range	Total	Male	Female
Number		228	109	119
Age		46.2 ± 9.9	49.2 ± 9.2	43.2 ± 9.7
Maslach Burnout Inventory scores				
Emotional exhaustion	(5–25)	12.4 ± 4.3	12.0 ± 4.3	12.7 ± 4.2
Depersonalization	(6–30)	11.8 ± 4.1	12.1 ± 4.2	11.5 ± 4.1
Personal accomplishment	(6–30)	20.2 ± 4.3	20.4 ± 4.0	20.1 ± 4.5
Devaluation-Discrimination score	(12–48)	30.9 ± 4.7	30.7 ± 4.5	30.9 ± 4.9

**Table 2:** Correlation between burnout and stigma.

	1	2	3	4
(1) Emotional exhaustion	—			
(2) Depersonalization	0.613^*∗∗*^	—		
(3) Personal accomplishment	0.266^*∗∗*^	0.273^*∗∗*^	—	
(4) Devaluation-Discrimination	−0.003	0.131^*∗*^	0.058	—

^*∗*^ *p* < 0.05; ^*∗∗*^*p* < 0.01.

**Table 3:** Hierarchical multiple linear regression analysis and the Devaluation-Discrimination scores on the Maslach Burnout Inventory.

	Emotional exhaustion		Depersonalization		Personal accomplishment	
	Step 1	Step 2	Step 1	Step 2	Step 1	Step 2
	*β*	*β*	*β*	*β*	*β*	*β*
Gender	−0.03	−0.03	−0.13	−0.14	−0.08	−0.08
Age	−0.23	−0.24	−0.10	−0.10	−0.16^*∗*^	−0.16^*∗*^
Devaluation-Discrimination		0.01		0.15^*∗*^		0.01
*R*	0.23	0.23	0.14	0.20	0.16	0.16
*R* ^2^	0.05	0.05	0.02	0.04	0.03	0.03
*R* ^2^ change score	0.05	0.00	0.02	0.02	0.03	0.00
*F*	5.72^*∗∗*^	3.80	2.07	2.96^*∗*^	2.73	1.82

^*∗*^ *p* < 0.05; ^*∗∗*^*p* < 0.01. Step 1: adjusted for gender and age; step 2: adjusted for Devaluation-Discrimination score.

## Data Availability

The data used to support the findings of this study are available from the corresponding author upon request.
